# Immunogenomic alterations of head and neck squamous cell carcinomas stratified by smoking status

**DOI:** 10.1002/ctm2.599

**Published:** 2021-11-06

**Authors:** Yixuan Huang, Peng Zhang

**Affiliations:** ^1^ The George Washington University Washington District of Columbia USA; ^2^ Beijing Key Laboratory for Genetics of Birth Defects, Beijing Pediatric Research Institute Beijing Children's Hospital, Capital Medical University, National Center for Children's Health Beijing CHINA; ^3^ University of Maryland School of Medicine Baltimore Maryland USA

**Abbreviations**
FDR: false discovery rate
GSEA: gene‐set enrichment analysis
HNSCC: head and neck squamous cell carcinoma
HR: hazard ratio
IFIT: interferon‐induced protein with tetratricopeptide repeats
OS: overall survival
TCGA: The Cancer Genome Atlas
TMB: tumor mutation burden
TME: tumor microenvironment
UMAP: uniform manifold approximation and projection

Dear Editor,

Accumulating evidence revealed that the immunologic sequelae of smoking may contribute to the progression and treatment resistance of various cancer types, including lung cancer and head and neck squamous cell carcinoma (HNSCC).^1^, ^2^ Moreover, according to the statistics on clinical outcome of immune checkpoint therapy, only a minority (10%–15%) of HNSCC patients could benefit from immunotherapy, and the smoking patients with HNSCC tend to have a lower response rate.^3^, ^4^ Numerous studies were aimed to access the single nucleotide variation and copy number aberration that was associated with tobacco use of HNSCC, but lacked immunogenomic investigation.^5^ Thus, there is urgently needed to examine the correlation between smoking and immunogenomic changes in HNSCC. Our results revealed a systemic effect of smoking on immune contexture of HNSCC and that would provide a roadmap for immunotherapy responsiveness prediction based on an immunological biomarker for future clinical usage.

To determine whether the tumor samples of smoking patients with HNSCC were enriched for high‐frequency mutations, we evaluated the somatic mutation profile of top10 most commonly altered genes based on The Cancer Genome Atlas (TCGA) database (Figure 1A). Interestingly, most of the high‐frequency mutated genes (9/10) had an increased number of somatic mutations in smoking HNSCC samples except for *CDKN2A* (Figure 1B). Besides, we found that the human papilloma virus (HPV)+ rate in smoking HNSCC (8.7%) was lower than that in the non‐smoking patients (20.9%) based on the TCGA database (Figure 1A), which may contribute to the immunological difference of tumor microenvironment (TME). Additionally, many studies investigated the relationship between smoking and prognosis and revealed the non‐smoking HNSCC patients tend to have a favorable outcome; however, the hazard ratio (HR) differed considerably among different studies.^6^ To get a more accurate assessment of prognostic significance, we compared the overall survival (5‐year) curve among different smoking statuses based on the TCGA HNSCC cohort. As shown in Figure 1C, the smoking HNSCC patients had a trend in poorer overall survival, but no substantial prognostic impact of smoking was observed (HR = 1.259, *p*‐value = 0.2338). Next, we compared the mutation count, single nucleotide variants (SNV) neoantigen, and the fraction of copy number variations to examine the effects of smoking on tumor immunogenicity. The smoking HNSCC patients showed a greater number of somatic mutations and neoantigens (Figure 1D). Interestingly, we did not observe a dramatic change in the percentage of copy number alterations between the smoking and non‐smoking HNSCC samples (Figure 1E), indicating the smoking made a major impact on gene mutations, but less on copy number variations in HNSCC. To gain insights into the molecular mechanisms underlying the functional impact of smoking on cancer cells of HNSCC, we analyzed the differential gene expression patterns between the tumor samples of the smoking and non‐smoking HNSCC (Figure 1F), smoking HNSCC samples and adjacent normal tissues (Figure S1A) and non‐smoking HNSCC samples and adjacent normal tissues (Figure S1B). The overlapping and specific differential expressed genes are shown in Figure S1C. A total of 549 up‐regulated genes and 1496 down‐regulated genes were identified in smoking HNSCC tumors. Gene ontology enrichment analysis of up‐regulated genes showed many cell‐cell signaling modules were significantly enriched in the smoking HNSCC samples (Figure 1G). Remarkably, lots of immune‐related gene ontology terms were enriched based on the down‐regulated genes, which specified the diminished immunological activity was observed in the tumor samples of the smoker with HNSCC (Figure 1G). We further performed gene set enrichment analysis (GSEA) and revealed that interferon‐mediated signaling signature scores significantly decreased in smoking HNSCC samples (Figure 1H). In summary, our results demonstrated a strong correlation between smoking and dysregulated interferon signaling pathways in HNSCC.

**FIGURE 1:**
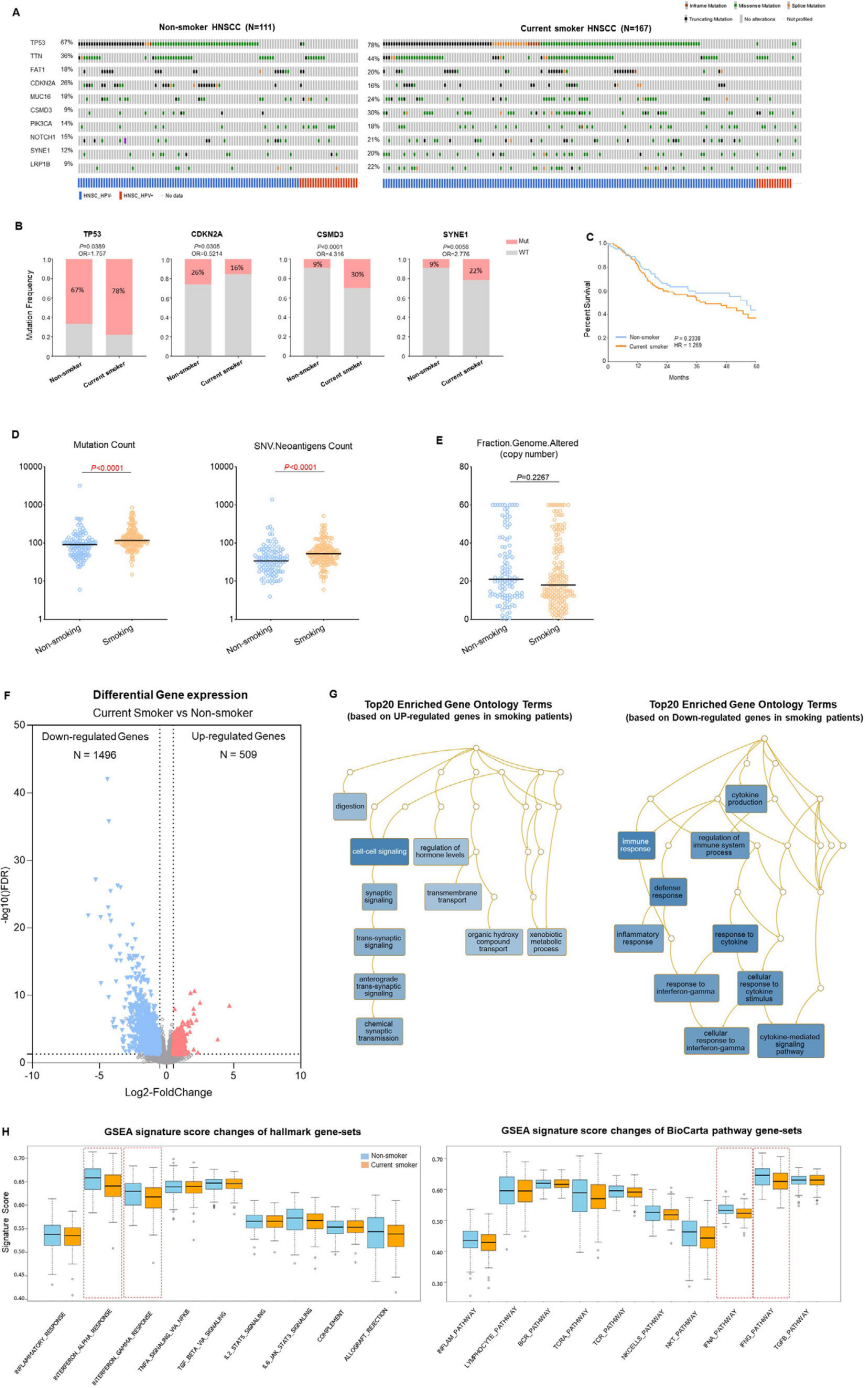
Effect of cigarette smoking on immunogenicity and immunological pathways in head and neck squamous cell carcinoma (HNSCC). (A) Distribution of total somatic mutation count among different smoking subtypes in the top 10 frequent altered genes of HNSCC. (B) Bar graphs showing the percentage of The Cancer Genome Atlas (TCGA) HNSCC samples with mutations in TP53, CDKN2A, CSMD3, and SYNE1 stratified by different smoking statuses. (C) Kaplan–Meier survival curve (5‐year) comparing the non‐smoker and current smoker with HNSCC based on TCGA database. (D) The dot plot showed the count of total somatic mutation (left) and SNV neoantigens (right) between current smokers and non‐smoker with HNSCC samples. (E) The distribution of the fraction of genome altered (FGA; the percentage of copy number altered chromosome regions out of measured regions) between current smokers and non‐smoker with HNSCC samples. (F) Volcano plot of mRNA expression changes between current smokers and non‐smoker samples with HNSCC. The x‐axis specifies the fold‐changes (FC), and the y‐axis specifies the negative logarithm to the base 10 of the adjusted *p*‐values. Gray vertical and horizontal dashed lines reflect the filtering criteria. Red and green dots represent genes expressed at significantly higher or lower levels, respectively. (G) Functional enrichment analysis (gene ontology) compared gene‐expression signatures based on the differential expressed genes. (H) The box‐plot showed gene‐set signature scores (MsigDB hallmark gene‐sets: left, and BioCarta pathway gene‐sets: right) among different smoking statuses. The solid line means the median value, and the two dashed lines mean the upper and the lower quartile of the data

To characterize the cell‐level difference of immunological response to tumors between smoking and non‐smoking HNSCC samples, we employed two cell‐deconvolution algorithms (CIBERSORT^7^ and XCELL^8^) to estimate infiltrating immune cell abundances based on the RNA‐seq profile of TCGA HNSCC dataset. As shown in Figure 2A,B, the smoking HNSCC samples had a significantly lower abundance of infiltrated activated NK and CD8 T cells. To confirm the specific immune phenotypes that were associated with smoking in HNSCC at a higher resolution, we accessed and reanalyzed a sophisticated single‐cell RNA‐sequencing (scRNA‐seq) dataset of HNSCC (nasopharyngeal carcinoma, including 10 non‐smoker and 5 smoker samples) with comprehensive infiltrated immune cell profiling.^9^ The unified expression profile of a total of 37 904 tumor‐infiltrating immune cells was compiled, and then 13 major immune cell clusters were identified and visualized (Figure 2C). The canonical markers used for the annotation of each cell type were shown in Figure 2D, and the cell lineage identification results between our analysis and the original report of the scRNA‐seq data were presented in Table S1. It is worth noting that we identified a unique subset of CD8T cells (CD8T‐IFI) that express high levels of interferon‐induced protein with tetratricopeptide repeats (IFIT) genes (Figure 2E). The statistical analysis showed the smoking HNSCC samples had a lower fraction of CD8T‐IFI, NK cell, and pDC, all of which were generally considered as interferons‐production cells (Figure 2F). Collectively, our results revealed that the smoking status of tumor samples was associated with reduced interferon‐induced immune cell infiltration in HNSCC.

**FIGURE 2:**
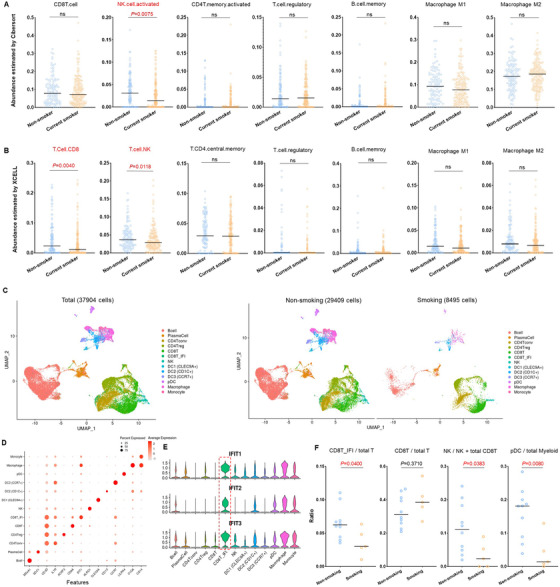
scRNAseq revealed specific immune cell types decline in smoker HNSCC samples. Distribution of immune cell abundance estimated by Cibersort (A) and XCELL (B) methods among different smoking subtypes in HNSCC. (C) Single‐cell RNA sequencing analysis of tumor‐infiltrating immune cells in HNSCC cancer (nasopharyngeal carcinoma). Uniformmanifold approximation and projection (UMAP) analysis showed 13 distinct clusters of cell phenotypes in the tumor microenvironment. (D) Dot plot of mean expression of canonical marker genes for immune each cell type. (E) Violin plot showed the expression of the markers for CD8T‐IFI (interferon induced protein with tetratricopeptide repeats) among different immune cell types in the TME of HNSCC. (F) Distribution of immune cell abundance derived from the scRNA‐seq dataset among different smoking statuses in HNSCC

Linking environmental factors to the immunologic activity and infiltrated immune phenotypes is of major interest in the field of immune‐oncology. The change of gene mutation and expression profiles among different smoking statuses have been explored extensively;^2^, ^5^ however, the effect of smoking on tumor immunogenicity and microenvironment in HNSCC has not been fully characterized. Although it is still difficult to conclude that smoking is a confounding or driving factor for the clinical outcome of HNSCC patients, the most noteworthy finding from our analysis is that smoking affects the abundance of some specific immune phenotypes in the TME of HNSCC, and all these altered infiltrated immune cells are interferon related. Thus, the smoking HNSCC samples exhibited significantly reduced interferon pathway activities and altered interferon‐producing immune phenotypes. Further prospective data with a large patient cohort will be needed to validate our results and definitively connect the smoking signature with the immunotherapy response of HNSCC in the future.

## CONFLICT OF INTEREST

Authors declare no competing financial interests.

## Supporting information

Supplement information

Supplement information

Supplement information
