# Supplementary figures and images for: Immunogenomic alterations of head and neck squamous cell carcinomas stratified by smoking status
Source: Clin Transl Med. 2021 Nov 6;11(11):1–5. doi: 10.1002/ctm2.599 (PMC8571948; doi:10.1002/ctm2.599)

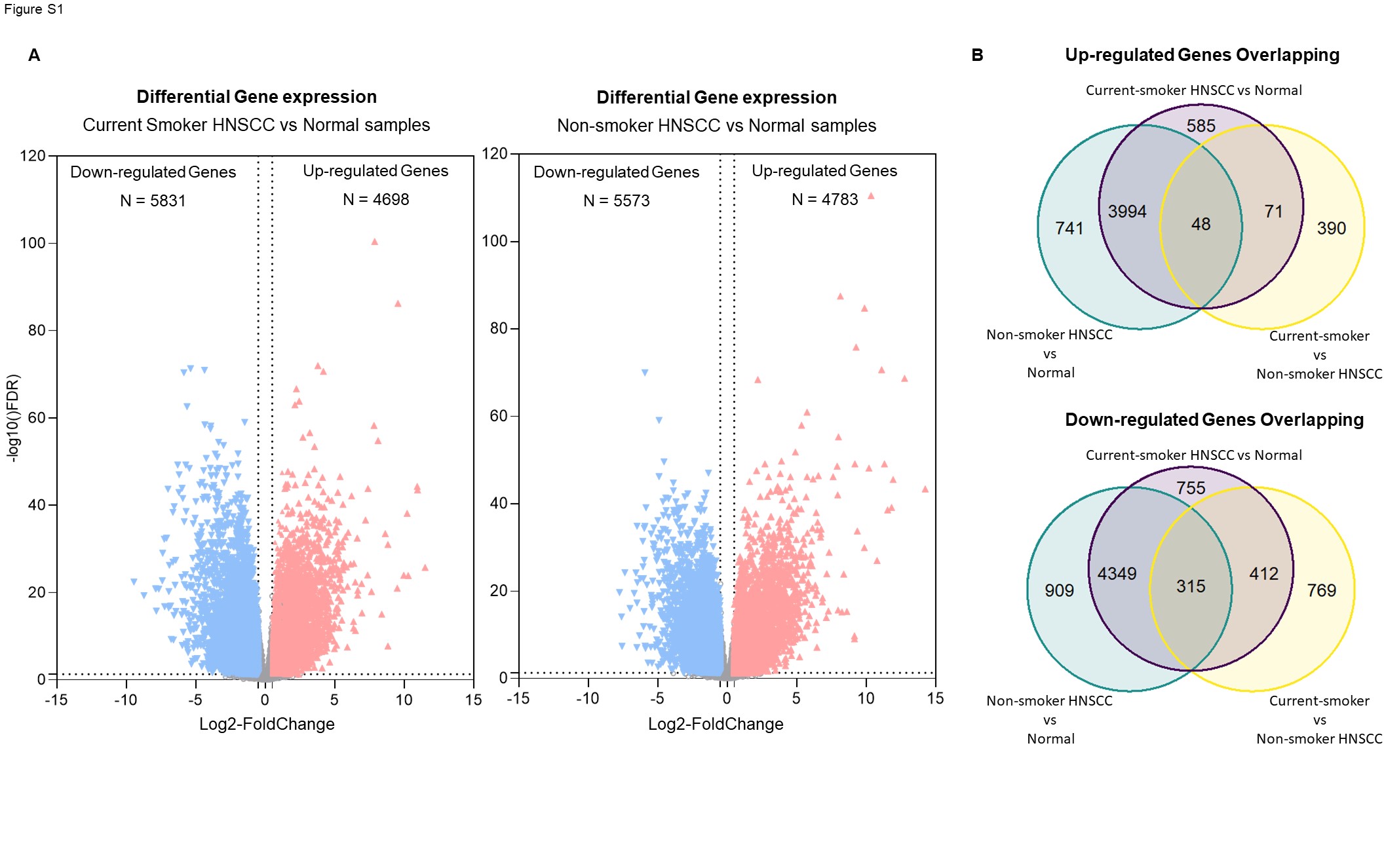

Supplement: Supplementary file 1 — Supplement information [file CTM2-11--s002.jpg]
