# Supplementary material for: Immunogenomic alterations of head and neck squamous cell carcinomas stratified by smoking status
Source: Clin Transl Med. 2021 Nov 6;11(11):1–5. doi: 10.1002/ctm2.599 (PMC8571948; doi:10.1002/ctm2.599)
Supplement: Supplementary file 2 — Supplement information [file CTM2-11--s003.docx]

**Supplementary Table 1. Comparison of identified cell types among different datasets**

| **Cell type specific**  **marker genes**  (shown in Figure 2D) | | **Cell type based on the reanalysis results in this work** | **Cell type identified in the original dataset**  (PMID: 32901110) | **Cell type in an independent sc-RNAseq dataset**  (PMID: 29198524) |
| --- | --- | --- | --- | --- |
| MS4A1 | B cells | B cells | B/plasma |  |
| SDC1 | PlasmaCell | PlasmaCell | B/plasma |  |
| CD3D, IL7R | CD4Tconv | CD4Tconv | CD4Tconv |  |
| CD3D, FOXP3 | CD4Treg | Treg | Treg |  |
| CD3D, CD8A | CD8T | CD8T, CD8Tdys | CD8T, CD8Texhausted |  |
| CD3D, CD8A, IFIT1 | CD8T_IFI | CD8T, CD8Tdys | CD8T, CD8Texhausted |  |
| KLRD1 | NK | NK | *Cells not profiled* |  |
| CLEC9A | DC1_CLEC9A+ | DC1 | *Cells not profiled* |  |
| CD1C | DC2_CD1C+ | DC2 | *Cells not profiled* |  |
| CCR7 | DC3_CCR7+ | DC3 | DC |  |
| LILRA4 | pDC | pDC | *Cells not profiled* |  |
| C1QA | Macrophage | Macrophage | Macrophage |  |
| CD14 | Monocyte | Monocyte | Macrophage |  |
