# Supplementary material for: Immunogenomic alterations of head and neck squamous cell carcinomas stratified by smoking status
Source: Clin Transl Med. 2021 Nov 6;11(11):1–5. doi: 10.1002/ctm2.599 (PMC8571948; doi:10.1002/ctm2.599)
Supplement: Supplementary file 3 — Supplement information [file CTM2-11--s001.docx]

**Supplementary Methods**

**Clinical Samples and Database**

We collected the largest publicly available cancer genomics database namely The Cancer Genome Atlas (TCGA) with genomic, transcriptomic, and clinical data. The clinical data and annotated mutation data of cancer samples were accessed from the cBioPortal for Cancer Genomics database (http://www.cbioportal.org/index.do, February 2019). The mRNA expression quantification profiles based on the RNA-sequencing were downloaded from the TCGA data portal (https://portal.gdc.cancer.gov/, February 2019). The extent of predicted SNV neoantigen count and the lymphocyte infiltration signature score of TCGA samples were accessed from “The Immune Landscape of Cancer” dataset in the NIH Genomic Data Commons web-server (https://gdc.cancer.gov/about-data/publications/panimmune, February 2019).

**Single-cell sequencing data analysis**

A sophisticated single-cell RNA-sequencing (scRNA-seq) dataset of HNSCC samples (nasopharyngeal carcinoma, including 10 non-smoking and 5 smoking samples) with the comprehensive tumor infiltrated immune cell profiling[1] was selected for the analysis of this work. The gene expression count profile was accessed from Gene Expression Omnibus (https://www.ncbi.nlm.nih.gov/geo/, GSE150430). Analyses were then performed using R software (https://www.r-project.org/, version 3.5) and primarily using packages “Seurat”. To process data, cells were filtered, keeping only those cells with the number of genes detected per cell > 300 and < 4000, and percent mitochondrial genes < 0.10. Samples were then log-normalized and scaled whereby numbers and two variables (unique molecular identifiers (UMIs) and percent mitochondrial genes) were regressed out. Clusters were determined by using the first 15 principal components and graphed by using the Uniform manifold approximation and projection (UMAP) dimensional reduction method for each sample. Each cluster was defined based on clustering and marker genes.

**Gene-set enrichment analysis**

Gene-set enrichment analysis (GSEA)[2] was performed using R software (https://www.r-project.org/, version 3.5) and primarily using packages “GSVA”. The hallmark gene sets provided by the Molecular Signatures Database (MSigDB, a collection of annotated gene sets for use with GSEA software) were selected for this analysis. The hallmark gene sets contained a total of 50 gene sets, which summarize and represent specific well-defined biological states or processes. The description and annotation (including the list of gene names) of these hallmark gene sets can be assessed by the MSigDB web-server (https://www.gsea-msigdb.org/gsea/msigdb).

**Biostatistical Analysis**

The specific tests used to analyze each set of experiments are indicated in the figure legends. For the comparison of the quantitative data between two groups, an unpaired Student's t-test with Welch's correction was used to calculate the p-value. To perform the differential gene expression analysis, the R package “DESeq2”[3] was employed to determine the significance of all genes. For the analysis of the contingency tables, Fisher's exact test was used to calculate the p-value. To perform a patient's cohort-based analysis on the overall survival rate (5-year), the prognostic value of discrete variables was estimated by using the Kaplan–Meier survival curves, and the log-rank test was employed to estimate the significance among different survival curves. All statistical calculations were performed using GraphPad Prism software (GraphPad Software, San Diego, California) or R software (https://www.r-project.org/).

**Availability of data or materials**

The materials of patient cohorts used for the current study were publicly available and can be assessed by the TCGA database (https://portal.gdc.cancer.gov/). The single-cell sequencing dataset[1] can be accessed from the Gene Expression Omnibus (https://www.ncbi.nlm.nih.gov/geo/) database with the accession number: GSE150430. The processed data and analysis codes are available upon reasonable request from the corresponding author.

Reference

1. Chen Y-P, Yin J-H, Li W-F, et al (2020) Single-cell transcriptomics reveals regulators underlying immune cell diversity and immune subtypes associated with prognosis in nasopharyngeal carcinoma. Cell Res 30:1024–1042. https://doi.org/10.1038/s41422-020-0374-x

2. Isakoff MS, Sansam CG, Tamayo P, et al (2005) Inactivation of the Snf5 tumor suppressor stimulates cell cycle progression and cooperates with p53 loss in oncogenic transformation. Proc Natl Acad Sci U S A 102:17745–17750. https://doi.org/10.1073/pnas.0509014102

3. Love MI, Huber W, Anders S (2014) Moderated estimation of fold change and dispersion for RNA-seq data with DESeq2. Genome Biol 15:550. https://doi.org/10.1186/s13059-014-0550-8
